# Synergistic *in vitro* activity of sodium houttuyfonate with fluconazole against clinical *Candida albicans* strains under planktonic growing conditions

**DOI:** 10.1080/13880209.2016.1237977

**Published:** 2016-12-08

**Authors:** Jing Shao, YanYan Cui, MengXiang Zhang, TianMing Wang, DaQiang Wu, ChangZhong Wang

**Affiliations:** a Laboratory of Microbiology and Immunology, School of Chinese and Western Integrative Medicine, Anhui University of Chinese Medicine, Hefei, China;; b Laboratory of Biochemistry and Molecular Biology, School of Chinese and Western Integrative Medicine, Anhui University of Chinese Medicine, Hefei, China

**Keywords:** Synergism, houttuynin, antifungal agent, β-1,3-glucan, resistance, ZAP1

## Abstract

**Context:** Fluconazole resistance is an intractable problem of treating *Candida albicans*, calling for more antifungal agents to enhance the activity of fluconazole.

**Objective:** This work investigates the anti-*C. albicans* activities of sodium houttuyfonate (SH) and/or fluconazole and the associated mechanism.

**Materials and methods:** The minimum inhibitory concentrations (MICs) of SH and fluconazole both ranging from 0.5 to 1024 μg/mL were determined by broth microdilution method in 19 *C. albicans* isolates, and their fractional inhibitory concentration index (FICI) was evaluated by checkerboard assay. After MIC_SH_ and/or MIC_fluconazole_ treatments, the expressions of *IFD6, PHR1, ZAP1*, *ADH5*, *BGL2*, *XOG1* and *FKS1* were analyzed by quantitative reverse transcription polymerase chain reaction (qRT-PCR) in *C. albicans* 1601.

**Results and conclusion:** The MICs of SH alone ranged from 32 to 256 μg/mL and decreased 2–16-fold in combination. SH showed strong synergism with fluconazole with FICI <0.13–0.5. In *C. albicans* 1601, we observed that (i) the expression of the seven genes increased notably in a range between 3.71- and 12.63-fold (*p* < 0.05) when SH was used alone, (ii) the combined use of SH and fluconazole slightly inhibited the expression of *IFD6* and *PHR1* by 1.23- and 1.35-fold (*p* > 0.05), but promoted evidently the expression of *ZAP1*, *ADH5*, *XOG1* and *FKS1* by 1.98-, 3.56-, 4.10- and 2.86-fold (*p* < 0.05). The results suggested SH to be a potential synergist to enhance the antifungal activity of fluconazole in *C. albicans* resistant isolates, and the underlying mechanism may be associated with β-1,3-glucan synthesis and transportation.

## Introduction

Recently, the incidence of invasive mycotic infection has increased significantly (Sardi et al. 2013). Among the infectious fungal pathogens, *Candida albicans* is the most frequently isolated opportunistic fungi from immunosuppressed individuals and patients implanted with types of catheters and artificial valves (Poulain 2013). Studies have shown that 75% of women were affected by vaginal candidosis at least once during their lifetime (Sobel 2007) and 90% of HIV-infected patients were suffered from oropharyngeal candidosis (de Repentigny et al. 2004). More importantly, invasive candidiasis was reportedly able to cause as high as 40–60% mortality rates (Tobudic et al. 2012).

Current antifungal agents include azoles, polyenes and echinocandins, among which azoles are the most common agent targeting the synthesis of fungal sterols (Zavrel & White 2015). As a consequence of increased abuse of traditional antifungal agents and antibiotics, the ever-increasing rate of resistance of *C. albicans*, especially to fluconazole poses a serious threat to antifungal therapy, calling for urgent need in search of novel antifungal drugs. To date, most of the reported chemicals claimed to possess potential antimycotic functions have relatively high minimum inhibitory concentrations (MICs); however, these antifungals usually had strong potential of resistance reversion in fluconazole-resistant *C. albicans* (Quan et al. 2006; Zhou et al. 2012; Letscher-Bru et al. 2013; Padmavathi et al. 2015). Therefore, finding new drugs capable of improving the antifungal activity of fluconazole can be taken into account as an alternative way to expand the antifungal bank (Guo et al. 2008).

Sodium houttuyfonate (SH, CH_3_(CH_2_)_8_COCH_2_CHOHSO_3_Na) is a chemical compound synthesized by houttuynin (CH_3_(CH_2_)_8_COCH_2_CHO) and sodium bisulfite (Shao et al. 2012). In previous reports, we observed antibacterial and antifungal potentials of SH against *Pseudomonas aeruginosa*, *Staphylococcus epidermidis* and *C. albicans* (Shao et al. 2013; Huang et al. 2015). Of interest, SH appeared to be more potent against *C. albicans* reference strain (MIC = 32–64 μg/mL) than *P. aeruginosa* reference strain (MIC = 256–512 μg/mL). To our knowledge, the antifungal effect of SH in combination with fluconazole and the underlying mode of action have not been reported.

The conventional mechanisms of resistance to fluconazole in *C. albicans* are usually attributed to overexpression/mutation of the target enzyme of azoles encoded by *ERG11* and drug efflux pump controlled by Cdr1p, Cdr2p belonging to ATP-binding cassette superfamily (APC transporter) and Mdr1p, a member of major facilitator superfamily (MFS) (Niimi et al. 2004; Holmes et al. 2008; Xiang et al. 2013; Prasad & Rawal 2014; Flowers et al. 2015). Nevertheless, a series of studies affirmed that sequestering fluconazole into cytoplasma caused by β-1,3-glucan, one of the main components in *C. albicans* cell wall and encoded by *FKS1*, accounted for fluconazole resistance in clinical *C. albicans* isolates (Mio et al. 1997; Nett et al. 2007; Zarnowski et al. 2014). Furthermore, β-1,3-glucan can be secreted into the supernatant that constituted the complex three-dimensional structure of *C. albicans* biofilm, also responsible for conferring fluconazole resistance to biofilm phenotype (Nett et al. 2007; Zarnowski et al. 2014).

In this study, we employed 18 *C. albicans* clinical isolates as well as a standard one to test the antifungal effects of SH and/or fluconazole via broth microdilution method, scanning electron microscope (SEM) and the expression of seven genes associated with β-1,3-glucan synthesis and transportation by quantitative reverse transcription polymerase chain reaction (qRT-PCR).

## Materials and methods

### Strains and cultivation

*Candida albicans* SC5314 was kindly provided by Prof. YuanYing Jiang from College of Pharmacy, Second Military Medical University, Shanghai, China. The clinical *C. albicans* isolates were kindly provided by Prof. HuaiWei Lu, Clinical Laboratory, Anhui Provincial Hospital, Hefei, China. These isolates were preliminarily identified by germ tube production, carbohydrate assimilation and fermentation by commercial Yeast Identification Kit Systems (Tianhe, Hangzhou, China). Further identification was performed by PCR method as described previously (Miyakawa et al. 1993). All strains were stored in YPD medium (1% yeast extract, 2% peptone, 2% dextrose; SHFENG, Shanghai, China) and 20% glycerol at −80 °C. After subculturing on sabouraud dextrose broth (SDB, SHFENG, Shanghai, China) for 24 h at 37 °C, these strain cells were harvested by centrifugation at 3000*g*, washed twice with sterile phosphate-buffered saline (PBS), resuspended in RPMI-1640 medium (Invitrogen, Carlsbad, CA), and calculated using a haemocytometer.

### Susceptibility test

The minimum inhibitory concentrations (MICs) of SH and fluconazole were determined by broth microdilution method based on CLSI M27-A3 (Clinical and Laboratory Standards Institute, 2008). The initial fungal cells were adjusted to 2 × 10^3^ CFU/mL in RPMI-1640 medium, and then added into a 96-well bottom-flat polystyrene microtiter plate. Both SH and fluconazole were serially two-fold diluted ranging from 0.5 to 1024 μg/mL and coincubated with the strain solution. The control contained fungal cells and broth medium, but with no drug. The MIC_90_ was defined as the lowest concentration of SH and fluconazole to cause 90% OD reduction at the wavelength of 490 nm by a spectrophotometer (SpectraMax M2/M2e, Silicon Valley, CA, USA) compared with the control. The checkerboard assay was used to assess the interactions of SH and fluconazole. The final concentrations of SH and fluconazole were serially 2-fold diluted in ranges of 2–128 and 0.25–256 μg/mL, respectively. The fractional inhibitory concentration index (FICI) was equal to (MIC_SH_ in combination/MIC_SH_ alone) + (MIC_fluconazole_ in combination/MIC_fluconazole_ alone), in which synergism was interpreted as FICI ≤ 0.5, indifference was defined as 0.5 < FICI ≤ 4.0, and antagonism was FICI > 4.0 (Odds 2003).

### qRT-PCR analysis

The procedures of qRT-PCR analysis were described in a previous study of our group (Shao et al. 2014). Briefly, 1 mL *C. albicans* 1601 strain broth (= 1 × 10^6^ CFU/mL) was mixed with 8 μg/mL fluconazole and/or 16 μg/mL SH at 37 °C for 24 h into a sterilized, flat-bottomed 24-well polystyrene microtiter plate (Corning, NY). The well with no agent was set as control. After centrifuging at 3000*g* for 5 min, the collected cell pellets were washed three times by sterilized PBS, and transferred into RNase-free screw-cap tubes. Total RNA was extracted by using MagExtractor-RNA kit (ToyoBo, Tokyo, Japan). Six microliters of the extracted total RNA was coincubated with 2 μL 4 × DNA Master: gDNA Remover and 2 μL 5RT-Master MixII. Then, the extracted RNA was reverse-transcribed into cDNA followed by: 65 °C for 5 min, 4 °C for 1 min, 50 °C for 5 min, 98 °C for 5 min and 4 °C for 1 min, according to ReverTra Ace qPCR RT Master Mix with gDNA Remover kit (ToyoBo, Tokyo, Japan). The 10-fold diluted cDNA was prepared before use. All experiments were performed on ice. The primers of *IFD6, PHR1, ZAP1*, *ADH5*, *BGL2*, *XOG1*, *FKS1* and *ACT1* (Table 1) were synthesized by Sangon Biotech (Shanghai, China). Real-time PCR mixture (= 25 μL) was composed of 12.5 μL 2 × SYBR Green Realtime PCR, 1 μL PCR Forward Primer, 1 μL PCR Reverse Primer, 0.5 μL cDNA and 10 μL ddH_2_O. The reaction was run on ABI7000 fluorescent quantitative PCR system (Applied Biosystem, Shanghai, China) with conditions as follows: initial step at 95 °C for 60 s, and then 40 cycles at 95 °C for 15 s, 55 °C for 15 s, 72 °C for 45 s. All data were normalized to housekeeping gene *ACT1* (the internal reference gene). The relative target-gene expression was calculated as a fold change of 2^−ΔΔCt^ value, in which ΔΔC_t_ = ΔC_t_^target gene^ − ΔC_t_^internal reference genes^ as previously described (Livak & Schmittgen 2001).

**Table 1.** The primers for qRT-PCR.

Primer	Sequence (5'-–3')
*IFD 6*-*f*	TTGGGAAGATTTTGATCCTGTTG
*IFD 6-r*	CGAGTGCATGATTTCTTCATAAGTG
*PHR 1-f*	CTGCAAAGCTGTTAGTGGAGTAGC
*PHR 1-r*	TGATGATCCAGAAGTAGATGCAGAG
*ZAP 1-f*	CGACTACAAACCACCAGCTTCATC
*ZAP 1-r*	CCCCTGTTGCTCATGTTTTGTT
*ADH 5-f*	GTTGGCCTTTGTCATTCAGAT
*ADH 5-r*	TACAAAGACCACATCCATTGGG
*BGL 2-f*	CTCGCAACTGTTCTTACTTCAGTTG
*BGL 2-r*	TGACGTCTTTACAAGTACCGTCATC
*XOG 1-f*	CAGTTGACGAATATCACTGGACA
*XOG 1-r*	AATATCCAACAATGGTTGACAGG
*FKS 1-f*	TGTGCTGGTCCAATGTTAGGATTATGTTG
*FKS 1-r*	TGAAACCTTCAGTGACCCACATAACAA
*ACT 1-f*	AGCTTTGTTCAGACCAGCTGATT
*ACT 1-r*	GGAGTTGAAAGTGGTTTGGTCAA

### SEM

After the treatments of SH and/or fluconazole, the sample was fixed by 2.5% glutaraldehyde overnight, and dehydrated by 30, 50, 70 and 100% ethanol for 10 min each. After air drying, the sample was sputter coated with gold in a vacuum evaporator, and the morphological observation was performed by a scanning electron microscope (SEM, JSM-6700F, Japan).

### Statistical analysis

All experiments were performed triplicate in three different occasions. The values were reported as mean ± standard deviation (SD) and calculated by SPSS 17.0 (SPSS Inc., Chicago, IL). One-way analysis of variance (ANOVA) was applied and *p* < 0.05 was considered as statistically significant.

## Results

### Antifungal activity of SH and/or fluconazole

**Figure 1.**
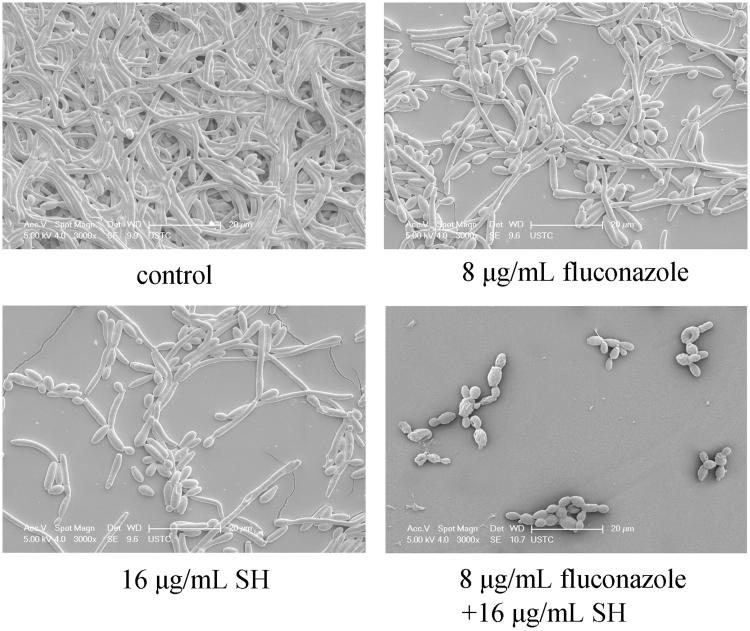
SEM inspection of SH and/or fluconazole against *C. albicans* 1601 when no drug, 8 μg/mL fluconazole, 16 μg/mL SH, and 8 μg/mL fluconazole and 16 μg/mL SH were used.

We employed 18 clinical *C. albicans* isolates as well as a reference strain *C. albicans* SC5314 to survey the antifungal effects of SH and/or fluconazole. It could be observed that the MICs of SH ranged from 32 to 256 μg/mL when SH was used alone, while decreased in a range of 8–64 μg/mL when used in combination with fluconazole. Compared with fluconazole used alone, the MICs of fluconazole were reduced 2–256-fold in concomitant use with SH. According to the FICI calculated, we found that SH was readily inclined to display synergism with fluconazole against fluconazole-resistant *C. albicans* isolates (MIC ≥64 μg/mL) in comparison with fluconazole-sensitive isolates (MIC <64 μg/mL, Table 2). Subsequently, the morphology was inspected by SEM in *C. albicans* 1601 at their synergistic MIC (8 μg/mL fluconazole and 16 μg/mL SH). Compared with the control, it was clear that the fungal cells were dramatically reduced and only yeast-form cells remained (Figure 1).

**Table 2.** Interactions of SH and/or fluconazole against clinical *Candida albicans* strains.

	MIC_90_ alone(μg/mL)		MIC_90_ in combination (μg/mL)		
C. albicans strains	SH	Fluconazole	SH	Fluconazole	FICI (Interpretation)
SC5314	32	1	16	0.5	1 (IND)
1601	128	128	16	8	0.19 (SYN)
1407	128	>1024	16	64	<0.19 (SYN)
1604	256	256	32	32	0.25 (SYN)
2009	256	1024	32	32	0.16 (SYN)
2209	256	>1024	32	4	<0.13 (SYN)
1110	256	32	64	16	0.75 (IND)
1107	128	512	8	64	0.19 (SYN)
1041	128	128	16	8	0.19 (SYN)
2305	64	32	32	8	0.75 (IND)
2301	128	256	64	8	0.53 (IND)
2103	128	1024	16	64	0.19 (SYN)
2304	256	>1024	32	64	<0.19 (SYN)
2111	64	>1024	16	8	0.25 (SYN)
2204	128	512	16	128	0.38 (SYN)
1803	64	128	16	32	0.5 (SYN)
2005	128	128	16	32	0.38 (SYN)
2226	64	>1024	16	64	<0.31 (SYN)
WY	128	4	32	2	0.75 (IND)

### Impacts of SH and/or fluconazole on gene expressions

**Figure 2.**
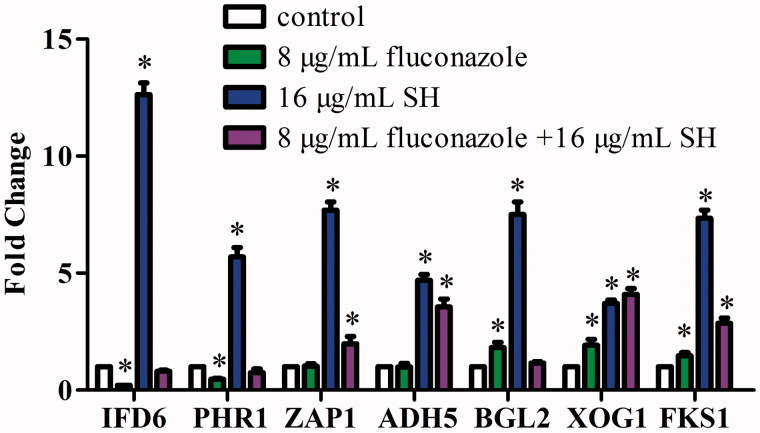
qRT-PCR analysis of *IFD6, PHR1, ZAP1*, *ADH5*, *BGL2*, *XOG1* and *FKS1* expressions under the treatments of no drug (control), 8 μg/mL fluconazole, 16 μg/mL SH, and 8 μg/mL fluconazole +16 μg/mL SH in *C. albicans* 1601. *p < 0.05, compared with the control.

**Figure 3.**
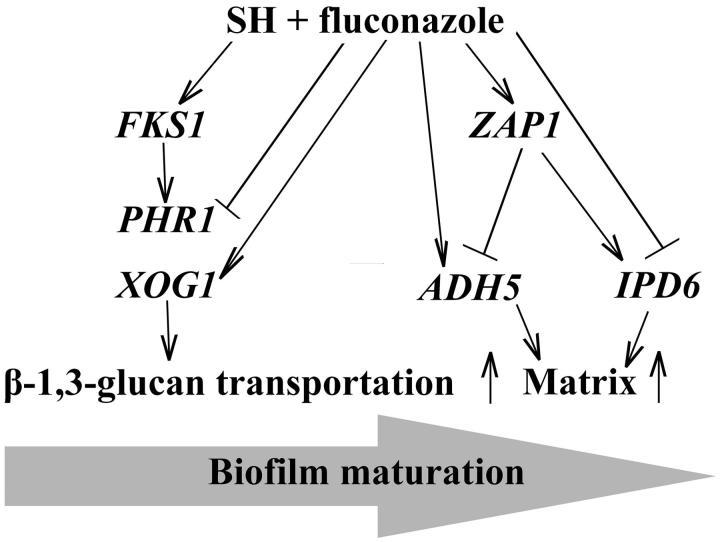
Illustration for the functions of SH and/or fluconazole on β-1,3-glucan transportation and biofilm maturation in fluconazole-resistant *C. albicans*.

Compared with the reference gene *ACT1*, the expression of *ZAP1* and *ADH5* was kept constant, while *IFD6* and *PHR1* was downregulated by 5-fold and 2-fold, respectively (*p* < 0.05), *BGL2*, *XOG1* and *FKS1* were upregulated by 1.82-, 1.92- and 1.47-fold after 8 μg/mL fluconazole treatment (*p* < 0.05, Figure 2). When exposed to 16 μg/mL SH, the expression of all tested genes exhibited notable increase in a range between 3.71- and 12.63-fold (*p* < 0.05, Figure 2). Under the combined application (8 μg/mL fluconazole +16 μg/mL SH), it could be observed that (i) *BGL2* was not affected, (ii) the expression of *IFD6* and *PHR1* was slightly inhibited by 1.23- and 1.35-fold respectively with no significant differences (*p* > 0.05), (iii) the expression of *ZAP1*, *ADH5*, *XOG1* and *FKS1* increased evidently ranging between 1.98- and 4.10-fold (*p* < 0.05, Figure 2). A simple illustration was presented to describe the effect of SH in combination with fluconazole on the gene expression associated with β-1,3-glucan transportation and biofilm maturation (Figure 3).

## Discussion

*Candida albicans* is currently the main cause for invasive fungal infections due to the recalcitrant resistance to traditional antifungal agents, such as fluconazole. Recruiting more drug-assisting fluconazole from the existent antibacterial and anti-inflammatory agents is a favourable option. SH was reported to possess mild antimicrobial activity against *P. aeruginosa* and *S. epidermidis*, and also show a certain inhibition on *C. albicans*. Due to limited isolates and experimental conditions adopted, however, we did not observe consistent MICs of SH against *C. albicans* (Shao et al. 2013; Huang et al. 2015). Herein, we expanded the *C. albicans* isolates and made a rigorous test on the antifungal activity of SH and/or fluconazole. SH alone displayed more efficient anti-*C. albicans* effect compared with its effect on pathogenic bacteria (Table 2). The synergism of SH with fluconazole against fluconazole-resistant *C. albicans* indicated the strong potential of SH to promote the therapy of fluconazole (Table 2). In addition, the haemolysis rate was less than 15% when the used concentration of SH alone reached to 256 μg/mL in a previous study of our group (Huang et al. 2015). As for *C. albicans* 1601, the cytotoxicity caused by the combined concentration of SH (= 16 μg/mL) can be negligible. Actually, we have injected 500 mg/kg SH (much higher dosage than that for clinical use) into 15 BALB/c mice, fed them for 90 days to evaluate their tolerance, and observed no death (Huang et al. 2015). However, we are trying an *in vivo* test to further evaluate whether SH can be a promising synergist in the treatment of fluconazole-resistant *C. albicans*.

As described previously, β-1,3-glucan in cell wall could nonspecifically interact with fluconazole to prevent from penetration into fungal cell (Nett et al. 2007), conferring partly a resistance to fluconazole in *C. albicans*. In this study, a group of genes associated with β-1,3-glucan synthesis and transportation were analyzed by qRT-PCR. As demonstrated previously, *ADH5* and *IFD6*, both of which are predicted to encode alcohol dehydrogenases, receive respectively negative and positive regulations of *ZAP1* encoding the zinc-response transcription factor Zap1 (Nobile et al. 2009). *ZAP1* mutant strain could promote the production of β-1,3-glucan by 1.5-2-fold in biofilm matrix than the complemented and reference strains with no significant difference in biofilm biomass (Nobile et al. 2009). *BGL2*, *XOG1* and *PHR1* were assumed in charge of three separate pathways of β-1,3-glucan transportation synthesized by *FKS1*, a distinct pathway out of the control of *ZAP1* (Taff et al. 2012).

In *C. albicans* 1601 (Figure 2), when fluconazole was used alone, the downregulated *IFD6* was a sign of the increase of β-1,3-glucan, consistent with the responses of *BGL2*, *XOG1* and *FKS1* (*p* < 0.05), inferring that the strain would produce and transport more β-1,3-glucan to the fungal cell wall and the outer space to sequester fluconazole. The transportation of β-1,3-glucan could be complemented by the upregulation of *BGL2* and *XOG1* when *PHR1* was inhibited. After SH was employed alone, the selected seven genes obtained notable expressions (*p* < 0.05). We presumed that SH might be able to suppress the accumulation of β-1,3-glucan outside the fungal cell by the negative regulations of *ZAP1* and *IFD6*. However, the concentration of SH (16 μg/mL) was not sufficient to inhibit the growth of *C. albicans* 1601, leading to more synthesis and transportation of β-1,3-glucan to the cell wall of growing fungal cells. Exposure to SH and fluconazole simultaneously could further significantly suppress the accumulation of β-1,3-glucan outside the fungal cell as *ZAP1* expression still acquired obvious increase (*p* < 0.05). To our surprise, the expressions of *ADH5*, *XOG1* and *FKS1* were significantly upregulated (*p* < 0.05). We hypothesized that SH could interact with β-1,3-glucan physically or chemically, inducing the enhancement of β-1,3-glucan synthesis and transportation.

In conclusion, we confirmed that SH could be a candidate of synergist with fluconazole against clinical *C. albicans* isolates. The qRT-PCR analysis of seven genes suggested the antifungal mechanism of SH and/or fluconazole was deeply involved with the synthesis and transportation of β-1,3-glucan.
